# Which stakeholders should be addressed to promote Geriatric Medicine among healthcare professionals, educationalists and policy-makers in European countries? – the PROGRAMMING COST 21,122 action experience

**DOI:** 10.1007/s40520-024-02841-4

**Published:** 2024-09-23

**Authors:** Sumru Savas, Nilufer Demiral Yilmaz, Marina Kotsani, Karolina Piotrowicz, Sofia Duque

**Affiliations:** 1 Department of Internal Medicine, Division of Geriatrics, Faculty of Medicine, Ege University, Izmir, Turkey; 2 Department of Medical Education, Faculty of Medicine, Ege University, Izmir, Turkey; 3 Hellenic Society for the Study and Research of Ageing, Agia Paraskevi, Greece; 4 Department of Internal Medicine and Gerontology, Faculty of Medicine, Jagiellonian University Medical College, Kraków, Poland; 5 Preventive Medicine and Public Health Institute, Faculty of Medicine, University of Lisbon, Hospital Cuf Descobertas, Lisbon, Portugal

**Keywords:** Geriatric medicine, Healthcare professionals, Stakeholders, Education, Training

## Abstract

**Background:**

Geriatric Medicine (GM), concerned with well-being and health of older adults, can play a crucial role in the alignment of healthcare systems to the needs of the aged populations. However, countries have varying GM development backgrounds. The goal of PROGRAMMING- COST 21,122 Action is to propose the content of education and training activities in GM for healthcare professionals across various clinical settings, adapted to local context, needs, and assets. Defining relevant stakeholders and addressing them on both an international as well as a country-specific level is crucial for this purpose. In this paper we are describing the methods used in the PROGRAMMING Action 21,122 to map the different categories of stakeholders to be engaged in the Action.

**Methods:**

Through conceptualizing a model for stakeholders by literature research, and online discussion group meetings, a synthesis for the potential stakeholders was defined as a template, and pilot applications were requested from participant countries.

**Results:**

There were 24 members from 14 countries (6 males/18 females) of multidisciplinary professions involved in this study. A model for the list of stakeholders to be addressed was developed and, after seven online discussion meetings, a consensus framework was provided. Invited countries completed the templates to pilot such operationalization.

**Conclusion:**

Our framework of stakeholders will support the research coordination and capacity-building objectives of PROGRAMMING, including the participation into the assessment of educational needs of healthcare professionals. Identified stakeholders will also be mobilized for purposes of dissemination and maximization of the Action’s impact. By defining and mapping multidisciplinary stakeholders involved in older people’s care specific to countries, particularly where GM is still emerging, GM tailored educational activities will be facilitated and optimally targeted.

**Supplementary Information:**

The online version contains supplementary material available at 10.1007/s40520-024-02841-4.

## Introduction

The world’s population is aging and ensuring that health systems will meet the needs of an aging population is crucial. As Geriatric Medicine (GM) deals with the health and well-being of older individuals, this field of medicine is critical to achieve this goal. However, not all countries have similar access to GM education, training facilities and resources so far [1]. In countries where GM is still developing, there is a huge demand not only for geriatricians but also for other healthcare professionals (HCPs) trained in GM principles for the care of older people [2]. Pragmatic solutions are required to deliver appropriate healthcare according to the needs of older people and optimize scarce specialized workforce resources. A feasible pragmatic solution can be GM tailored education and training of the existing workforce, which is affordable, exponentially efficient, and, thus, highly relevant. Such tailored educational activities should be both compatible with GM principles but also respecting country specific characteristics and needs. Fulfilling those requirements, and reaching those goals necessitate interdisciplinary network collaboration within and across countries and optimization of assets.

In this context, the aim of the “European Cooperation in Science and Technology (COST) Action CA21122 - PROmoting GeRiAtric Medicine in countries where it is still eMergING” (PROGRAMMING) is to reach to a consensus about the content of targeted education and training activities in GM for HCPs across various clinical settings. PROGRAMMING also aims to raise awareness and promote the added value of the specialized approach of GM for the health and wellbeing of older people among HCPs, policy makers, older people and the general public. It will address mainly countries where GM is still emerging, adapted to the local context, the needs and assets of stakeholders and the pragmatic possibilities of involved settings (https://www.cost.eu/actions/CA21122/*)*, (https://www.cost.eu/), (www.cost-programming.eu) [3–5]. It engages a network of more than 300 individuals from more than 40 countries, including geriatricians, doctors of other medical specialties, dentists, nurses, physiotherapists, nutritionists/dieticians, pharmacists, occupational therapists, psychologists, gerontologists, academics (educationalists and researchers), other allied HCPs, IT researchers, and others.

Dissemination of PROGRAMMING results and proposed solutions to stakeholders, policy makers and the public is one of the key missions of Working Group 5 of the Action. Identification of national and international stakeholders to address is therefore a priority task to be undertaken.

In order to succeed in communicating and disseminating PROGRAMMING messages and reach our goals, the audience and stakeholders we wish to engage will be much broader than only HCPs and therefore must be carefully and strategically identified. Indeed, we must consider that well-being and quality of life of olders persons do not depend only on the healthcare, but also on social, financial, functional, environmental, and cultural dimensions. Regulatory bodies in these fields, both at the national and international levels, must be aware of and address the challenges associated with aging. They should also recognize the significance of GM as an interdisciplinary discipline that extends beyond specialized healthcare services.

The literature on identifying relevant stakeholders about GM education promotion is scarce and setting a frame applicable across several countries with different education and health structure organisation can be challenging. In a recent study exploring the perspectives of Gerontology stakeholders on healthy aging and the readiness for a healthy aging society, the authors included various participants such as practitioners, care providers, non-governmental organizations, policy makers, and researchers, aside from geriatricians [6]. As older individuals have both health problems and functional impairments, the care of older people with complex issues typically necessitates integration of health and social services, and different countries might have different programmes [7], and substantial structural differences in their health and social care systems. The integration of health and social services might be at levels such as organizational, professional, system or service [7]. Besides, interprofessional education and collaborative practice (IPECP) [8] that begin with education and extends to continuous professional development involving various players [8–10] might be useful for designating GM stakeholders conceptualized in macro (governance/system) and meso levels (institutional/local) regarding education and labor sectors [8, 9]. As PROGRAMMING audience and stakeholders to reach are so wide and distinct, a discussion was held among the Action members to list the different categories of stakeholders to be approached. In this paper we are describing the methods used in the PROGRAMMING COST Action to map the different categories of stakeholders to be engaged.

## Methods

**Fig. 1:**
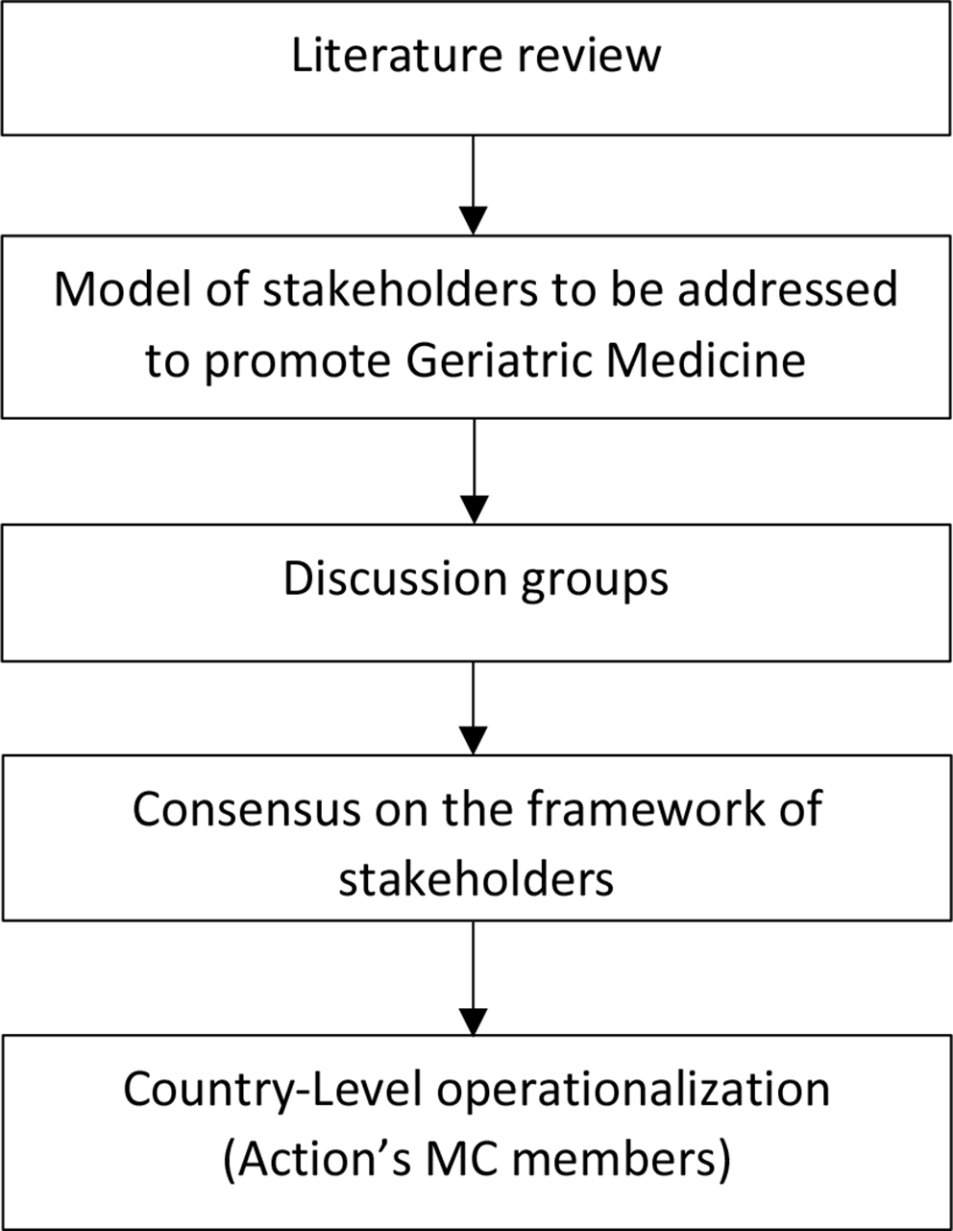
Flowchart of methodology *MC, Management Committee

The methodology of our approach is summarized in the flowchart of Fig. 1.

A literature search was perfomed in PubMED database, looking for manuscripts including information about various group of stakeholders deemed significant to foster GM. The keywords used were: Geriatric Medicine, education, stakeholders, healthcare professionals, educationalists, policy-maker, interprofessional education, integrated care, and law. The abstracts of the articles, and also their references were reviewed.

**Fig. 2:**
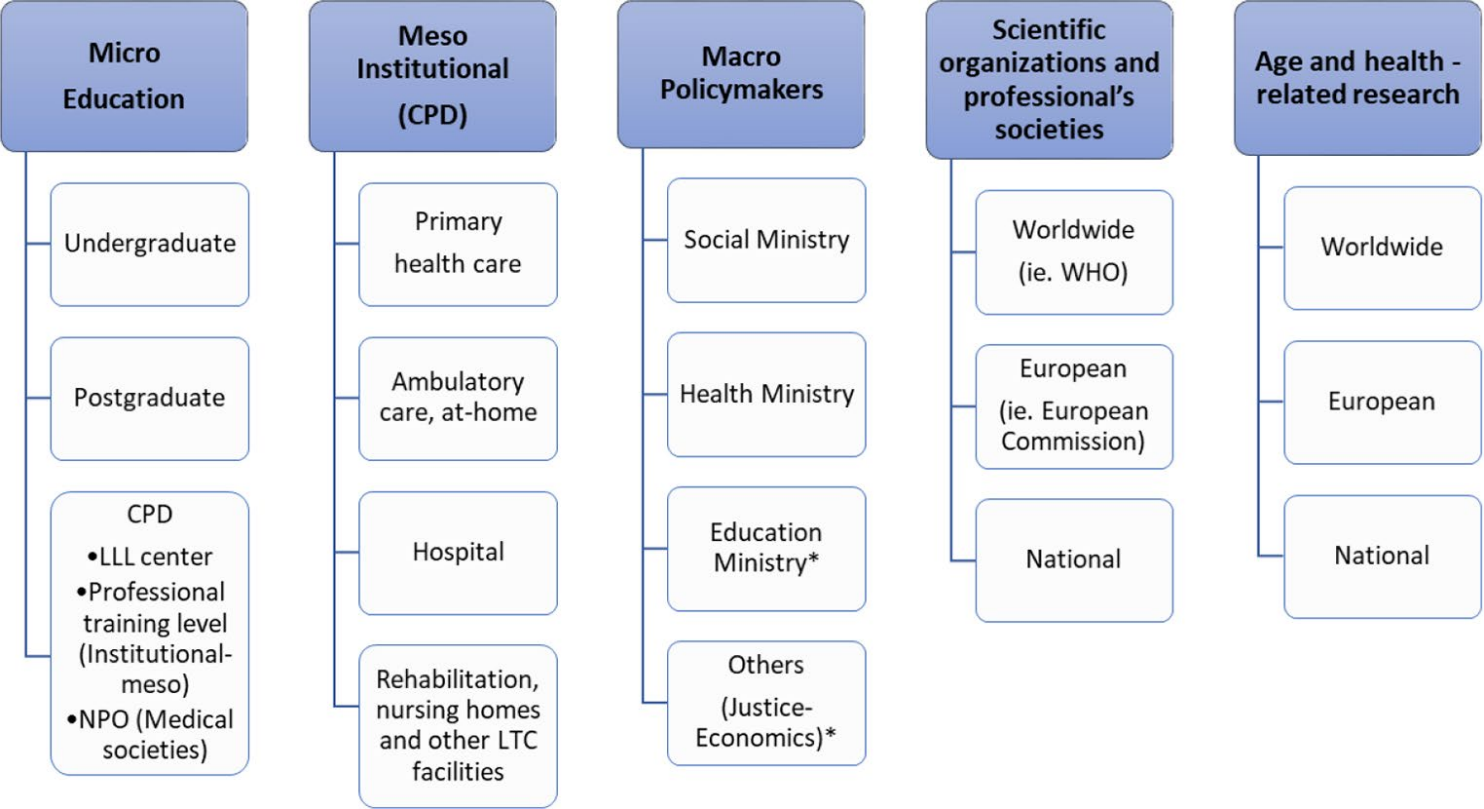
Model of stakeholders to be addressed to promote Geriatric Medicine *May differ according to countries CPD, Continuous Professional Development; LLL, Life Long Learning; NPO, Non-Profit Organizations; LTC, Long Term Care; WHO, World Health Organization

Following the literature review in combination with expert opinions (SS, NDY, SD, KP, MK), a possible level-based model to categorize stakeholders to be addressed in the scope of PROGRAMMING was prepared. Microlevel stakeholders concern education providers, from undergraduate to continuous professional development. Mesolevel stakeholders cover healthcare facilities or providers delivering care to older persons of different settings - community (primary care, ambulatory, at-home), hospital and rehabilitation, nursing homes and long-term care facilities. Macrolovel stakeholders encompass governance bodies such as Health, Social and Educational Ministries. World Health Organization is a major international macrolevel stakeholder, as well as European Union bodies related to health and ageing policies. National and international organizations, societies and research bodies related to ageing, Geriatrics and Gerontology are also included in this model (Fig. 2) [10]. This model was derived and amended from perspectives on integration across models for care of older people with complex needs and IPECP concepts [7–9].

After the conceptualization of the stakeholders’ framework (Fig. 2), PROGRAMMING Working Group 5 members were invited to reflect and share their input on the stakeholders to reach, considering both national and international levels. In compliance with the COST Action principles, discussion groups were formed, ensuring diversity and inclusiveness, through joining participants from different countries, professional backgrounds, age and gender. We expected participants of the discussion groups to provide different and complementary perspectives during online meetings.

Online meetings started with an explanation of the goal of the activity by the moderator of the discussion group (SS). The stakeholders framework (Fig. 2) was introduced and participants were asked to comment according their personal experience, both national and international level. Participants were invited to validate, operationalize and adapt the stakeholders framework to their own national context.

To guide the brainstorming, a script of questions was used by the activity moderator (SS) to faciliate and harmonize the discussion groups (Table 1). The participants were encouraged to start discussing a set of questions that were expected to inspire the exchange of ideas and experiences and help us in identifying relevant specific stakeholders for the four years of the Action.

**Table 1:** Discussion groups guide

1	Who are the potential stakeholders we should reach in order to promote Geriatric Medicine in PROGRAMMING affiliated countries? Take into account potential stakeholders related to all healthcare professionals’ categories (e.g. medical students, medical doctors, dentists, nurses, pharmacists, social workers, dieticians, physiotherapists, psychologists, speech language/occupational therapists, nurse/healthcare assistants, dental technicians, any other allied healthcare professionals (HCPs).
2	With regard to **Geriatric Medicine education**, who are the stakeholders relevant to HCPs involved in care of older persons specifically in your country? And who should be contacted in your country, and how? Please, consider various categories of education, including: *Undergraduate education*,* Postgraduate education* (Master & Doctorate, Specialization), *Continuous Professional Development* (continuous medical education & professional training level) of *all the allied workforce* working in the field; if there is an inclusive institution covering most of the HCPs, please indicate.
3	With regard to **policymaking**, who are the potential stakeholders in relation to HCPs and other professionals involved in care of older persons (e.g. social sector professionals), specifically in your country? Who are the stakeholders responsible for setting policies? Which official institutions have a role in policymaking in relation to ageing and older adults in your country, if any?
4	Which ageing research institutes or societies could we contact (in relation to researchers)?
5	Which World / European / national scientific organizations, societies, Non-Governmental Organizations could we contact? ***How to reach ****them ****specifically at country-level?*** Specify in your country.
6	Provide details of the **stakeholders you have identified** (name of organization, contact details and website).

All the suggestions were discussed and critically appraised by discussion groups members before being considered to be included in the framework. Whenever a new stakeholder was mentioned, it was discussed within the group and, if consensually accepted by the majority of the group, it was included in the framework. The category if each stakeholder was also decided by agreement between discussion groups attendees.

After incorporating the output of all the discussion groups, a consensus framework of stakeholders was delivered (Table 2).

**Table 2:** Categories of stakeholders – a possible framework

A. EDUCATION		
Undergraduate	Postgraduate	Continuous Professional Development / Education
*Possible stakeholders*: Universities, including the Faculties of Medicine, Nursing Sciences, Psychology, and others	***1. Master & Doctorate.****Possible Stakeholders*: Universities, including Health Sciences Faculties and various Institutes	***1. In-Service Training (institutional)*** ***2. Life-long learning Centers. 3. Non-Profit Organizations****Possible Stakeholders*: Universities, Health/ Social/ Education ministries, Non-Profit Organizations, governmental commission, Professional organizations etc.
	***2. Specialization. ****Possible stakeholders*: Universities, Health Ministry, governmental specialization commissions.	
**B. HEALTH SYSTEM**,** EDUCATION**,** SOCIAL POLICY MAKERS (International / National) (including Justice / Economy Ministry**,** City councils**,** WHO**,** EU bodies)**		
**Ministry of Health**	**Ministry of Social Services (or related)**	**Ministry of Education**
**C. ORGANIZATIONS**,** SOCIETIES**,** EDUCATIONALISTS (International / National)** Eg: Non-Governmental Non-Profit societies, scientific societies, educational societies, professional societies, student organizations, charities		
**Labor Unions**,** Medical Chambers**	**Community**	**Scientific / Educational Organizations, Societies**
Of: Medicine, Dentistry, Nursing, Pharmacy etc.	Senior organizations, charities	Professional scientific societies, student’s organizations, Deans
**D. RESEARCH (International/ National)**		
Eg: World / European and national Geriatric / Ageing / Gerontology societies, social sciences / Health science / Public health - ageing research institutes, research funding organizations etc.		

*EU, European Union; WHO, World Health Organization

The final step involved an initiative to operationalize the consensus framework of stakeholders across different countries. To pilot such operationalization, participants of the discussion groups were invited to apply the consensus framework to their national contexts.

## Results

In the literature review, we found there was no article directly aiming to address all stakeholders for GM promotion in a holistic way among all possible HCPs, educationalists, policy-makers, and organizations. We found some limited data regarding specific contexts, such as integrated care, IPECP [7–9], long-term care [11], community-based geriatric healthcare workforce [12], online interdisciplinary Gerontology education [13]. In a recent systematic review of research barriers, facilitators, and stakeholders in long-term care and geriatric settings, and a conceptual mapping framework to build research capacity; 12 stakeholders were identified, and a final conceptual framework was generated (Research team, residents and patients, caregivers, staff, facilities and centers, ethics review committees, managers, management and regional authorities, research networks and groups, funding agencies and institutes, foundations, and trainees) [11]. Another study addressed the key stakeholders for the community-based geriatric healthcare workforce by the potential of interprofessional teams [12]. A developmental process model was introduced for online interdisciplinary Gerontology education identifying internal and external stakeholders with a holistic approach [13].

Twenty four members from 14 countries (6 males and 18 females) from different professional backgrounds participated in the discussion groups online meetings. Represented countries were: Albania, Bosnia and Herzegovina, Croatia, Cyprus, Estonia, Greece (four members), Kosovo, North Macedonia (two members), Poland, Romania, Serbia (three members), Spain, Switzerland, and Türkiye (five members). Seven online discussion meetings were held. Represented working fields were: medical college, surgery, radiotherapy and oncology, oncology, public health, pulmonary disease, pharmacy, dental medicine, internal medicine, gerontology, hygiene and tropical medicine, geriatrics, palliative medicine, community medicine, ophthalmology, and physical and rehabilitation medicine. The group also included a media analyst, head project manager, and a doctor of philosophy. Therefore, the groups consisted of medical doctors from hospital settings and community, researchers and professors (assistants, associate and full professors) from different backgrounds and professions. Participants from countries with well-established GM systems contributed with their experience and know-how on which stakeholders categories must be included in the ideal network of providers of care.

The discussion groups and the participants can be seen in supplementary material 1 (S1).

Table 2 operationalises the synthesis of the literature review and discussion groups. In this template potential stakeholders are defined both horizontally and vertically (Table 2). We decided to include both the generic name of the group of stakeholders and specific examples with contact details in a structured format.

In the discussion groups, there was a consensus that education was a key area, with subdivisions such as undergraduate, postgraduate, and continuous professional development. Addressing stakeholders related to continuous professional development was considered a priority, as most HCPs dealing with older patients lack training in GM.

Policymakers in education, health, and social systems were collectively deemed very important and recognized as key stakeholders by consensus. This included primary entities such as the Ministry of Education, the Ministry of Health, and the Ministry of Social Services (or equivalent). The chambers and professional scientific societies were considered substantial, particularly for workforce training, and were categorized under organizations, societies, and educationalists. Finally, research institutes and organizations were all recognized as key stakeholders. Defining different stakeholders other than readily present in the model was very difficult in the discussion groups.

The consensus framework of stakeholders was applied by members from Poland, Switzerland, Albania, Slovakia, Türkiye, Romania, Kosovo, Estonia, Serbia, and Norway. Their operationalization framework was shared as an example for replication by other countries. These frameworks have already proven useful for research activities within the scope of the Action.

## Discussion

Through a synthetic approach involving literature review, we have developed a model template for identifying multidisciplinary stakeholders relevant to the care of older people and related policymaking, applicable both nationally and internationally. Although no articles specifically aimed at addressing stakeholders for the promotion of GM among HCPs, educators, and policymakers were found in the literature search, some linked articles were identified. By gathering input from discussion groups, a consensus framework of stakeholders was obtained.

**Fig. 3:**
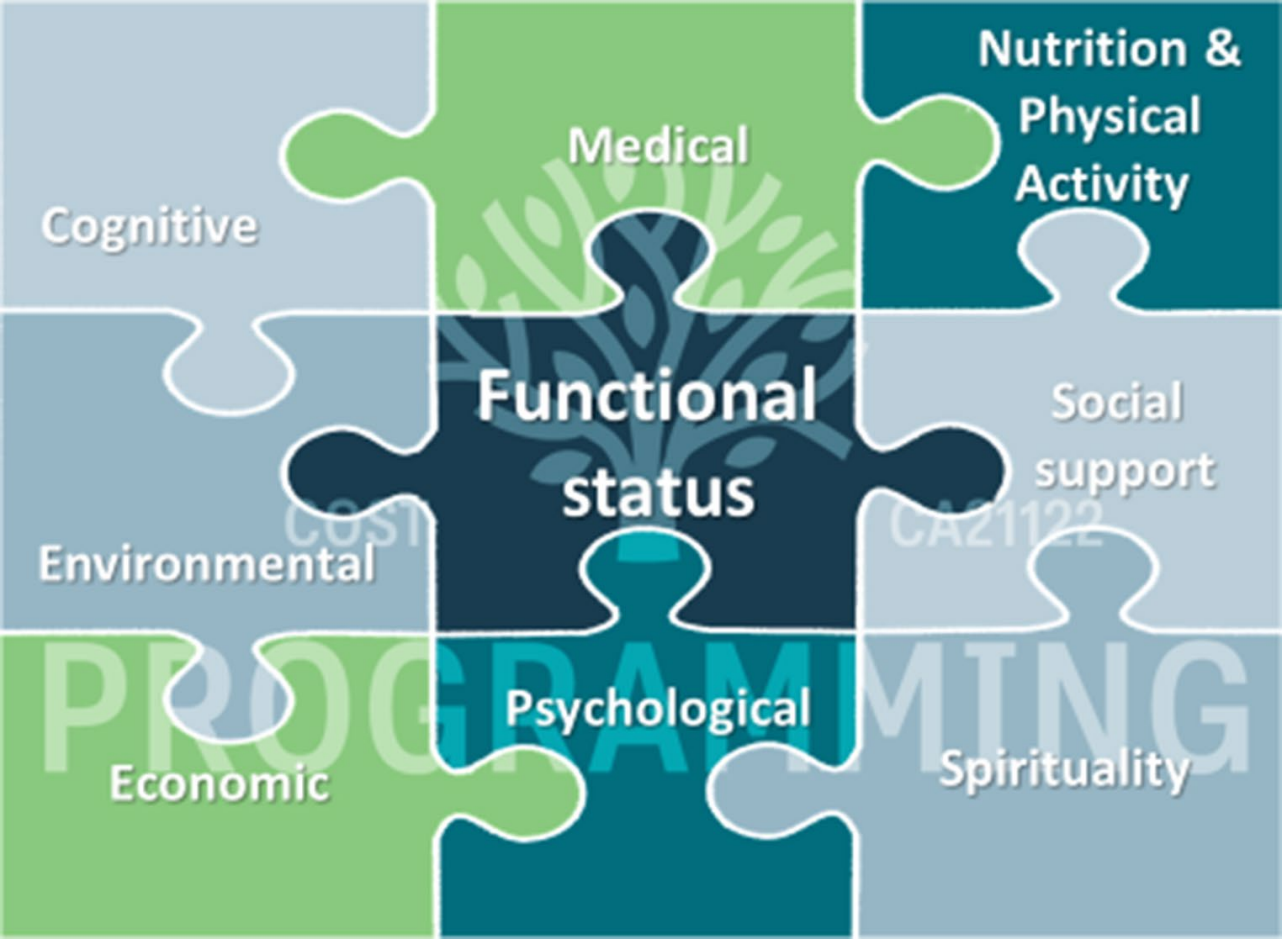
Domains of comprehensive geriatric assessment

To identify these stakeholders it is crucial to understand the specificities of GM. Geriatric Medicine encompasses the Comprehensive Geriatric Assessment, which involves a thorough evaluation of an older individual’s physical, mental, and social well-being making up the functional status of the individual comprised of medical, cognitive, psychological, socio-economical, and environmental domains as well as nutrition and physical activity (Fig. 3).

**Fig. 4:**
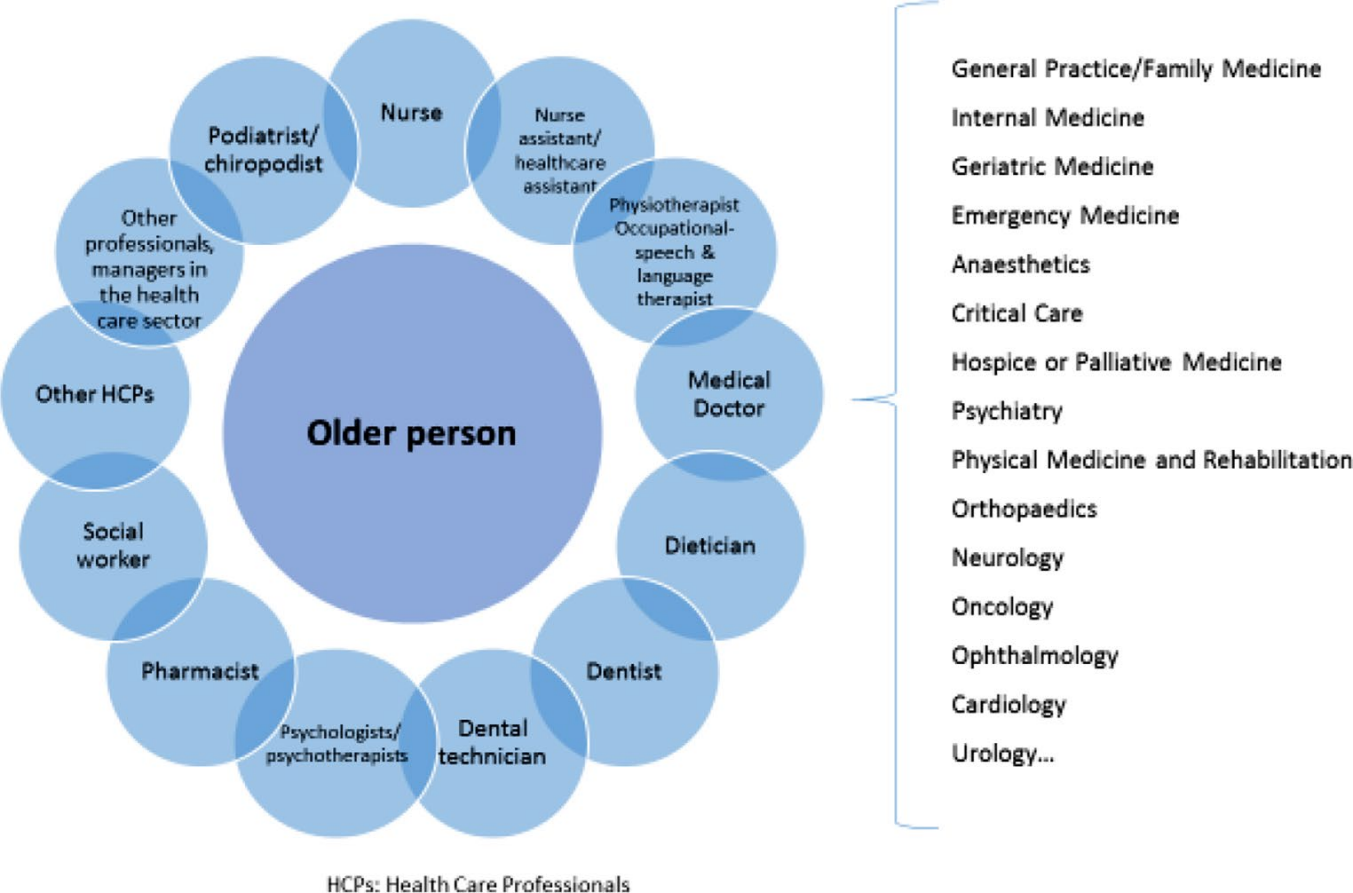
Healthcare Professionals involved in older persons care *HCPs, Healthcare Professionals

This assessment is conducted by a multidisciplinary team consisting of HCPs from various disciplines, including but not limited to physicians, nurses, psychologists, physiotherapists, dietitians, and social workers (Fig. 4). The team collaborates to develop a holistic treatment and follow-up plan that addresses the unique needs and challenges faced by older adults, aiming to preserve their functionality and improve their overall quality of life. It is therefore clear that the practice of GM and the need of specialized training is not limited to geriatricians. Given the demographic trend of the population wordwide, indeed most HCPs at some point will deal with older patients; therefore, most HCPs need basic competences in GM.

Given the multidisciplinary nature of GM, stakeholders involved in older persons care and advocacy for GM are very broad and its categorization is a challenging task, with national and international variations.

Remarkably, some of the countries involved in the Action do not officially recognize GM as a medical specialty or subspecialty, nor do they structurally incorporate specialized geriatric care services into their national health and social care systems. In such scenarios, the stakeholders to engage with may differ. This applies both to ‘strategic’ stakeholders with policy-making ability and to the ‘first line’ stakeholders, the professionals that find themselves principally caring for older people in everyday clinical practice in the absence of geriatricians. Additionally, this reality could impact interprofessional communication, hinder the recognition of geriatric medicine, and limit the establishment of effective networks among allied health professionals, which makes awareness of basic principles of GM even more relevant for all actors involved in the care of older people.

During the discussion groups we case studied different national experiences and examples, that were useful to reach a consensus framework of stakeholders. We also tested the operationalization of this framework for different countries.

One of the biggest challenges in building the consensus framework of stakeholders is the diversity among countries in terms of GM education and development, as well as their healthcare and social systems.

Since the aim of this study was not to identify the differences in education, organization, and policies of the countries regarding Geriatrics, but rather to focus on the methodology used in general and specific stakeholder analysis, a detailed summary for each country was not thoroughly specified in this article. However, some examples of differences at various levels, such as educational, organizational and GM background, are mentioned.

Participants of the discussion groups started mentioning that GM is still not recognized as a medical specialty in some European countries, such as Albania, Greece and Portugal. Great variety of education systems was found amongst countries that were represented in the discussion groups. For example, in Albania, doctors, dentists, pharmacists, nurses and other HCPs are required to attend accredited activities for continuous professional developmentto maintain their licenses. In Türkiye, higher education institutions such as universities are affiliated with organizations like the Higher Education Institution, which is directly linked to presidency. In Kosovo, all education programs have to be accredited by Agency of Accreditation.

Concerning policymaking bodies there is also significant diversity: for example, Estonia does not have Ministry of Health and health related research projects are under the responsability ofthe Ministry of Education and Research.

Our approach to identifying relevant stakeholders has both strengths and limitations. We have developed a consensus framework of stakeholders to address when fostering GM in various clinical settings. This framework has already been successfully used in mapping stakeholders at the national level to answer an online survey about educational needs in GM. It was first operationalized by some countries represented in the discussion groups, and their outputs were shared with other countries participating in PROGRAMMING. This process facilitated the creation of stakeholder maps in other countries.

Regarding the limitations of our approach, we did not conduct a systematic review of the literature on stakeholders. Additionally, a limitation of the discussion groups is the lack of representation from all European countries and all professional backgrounds related to the care of older persons, which may limit the comprehensiveness of stakeholder categorization. Finally, discussion groups did not follow the strict methodology of focus groups and qualitative studies.

## Conclusions

PROGRAMMING COST 21,122 Action aims to raise awareness and promote the added value of the specialized approach of GM for the health and wellbeing of older people among HCPs, policy makers, older people and the general public. Identifying all the key players to foster the PROGRAMMING mission is a demanding and challenging task, but can be decisive in attaining the goal of developing GM in countries where it is still emerging. This framework benefited from the input of members with multidisciplinary backgrounds from different countries, and will support the research coordination and capacity-building objectives of PROGRAMMING, as well as the dissemination and maximization of the Action’s impact.

The consensus framework of stakeholders can promote and facilitate the cooperation and coordination between stakeholders of different categories. We believe it can also inspire and maximize the impact of other future initiatives related to the promotion of GM for research aims, dissemination, communication, and networking activities. Indeed, a long-term goal of PROGRAMMING is to influence policymakers at the local level and the international scene in favor of developing specialized geriatric care for older people, with the aspiration to obtain at least a basic level of services throughout all involved countries.

The operationalization of this framework in different countries is expected to yield some differences, given the varying levels of development in GM education and clinical practice.

The identification of relevant stakeholders is a crucial step for communicating this message and for building collaborative synergies for meaningful clinical practices and impact maximization. PROGRAMMING aspires to hand over this legacy.

## Electronic supplementary material

Below is the link to the electronic supplementary material.

Supplementary Material 1

## Data Availability

No datasets were generated or analysed during the current study.
