# Supplementary material for: Which stakeholders should be addressed to promote Geriatric Medicine among healthcare professionals, educationalists and policy-makers in European countries? – the PROGRAMMING COST 21,122 action experience
Source: Aging Clin Exp Res. 2024 Sep 23;36(1):194. doi: 10.1007/s40520-024-02841-4 (PMC11420320; doi:10.1007/s40520-024-02841-4)
Supplement: Supplementary file 1 — Supplementary Material 1 [file 40520_2024_2841_MOESM1_ESM.docx]

**Supplementary material 1 (S1)**

**Discussion groups**

1. Meeting 1 (03/07/2023)

Türkiye (3), Romania (1), Serbia (2), Greece (2), Crotia (1)

2. Meeting 2 (03/08/2023)

Türkiye (3), North Macedonia (1), Cyprus (1), Bosnia and Herzegovina (1), Serbia (1), Albania (1), Greece (1), Kosovo (1)

3. Meeting 3 (03/24/2023)

Türkiye (1), Albania (1), Bosnia and Herzegovina (1), Kosovo (1)

4. Meeting 4 (03/27/2023)

Türkiye (2), (Greece (3)

5. Meeting 5 (03/28/2023)

Türkiye (1), Poland (1), Estonia (1), Serbia (2)

6. Meeting 6 (03/29/2023)

Türkiye (1), Cyprus (1), North Macedonia (1), (Romania (1), Greece (1)

7. Meeting 7 (03/31/2023)

Türkiye (3), Spain (1), Switzerland (1)
